# Supplementary material for: Superior photo-carrier diffusion dynamics in organic-inorganic hybrid perovskites revealed by spatiotemporal conductivity imaging
Source: Nat Commun. 2021 Aug 18;12:5009. doi: 10.1038/s41467-021-25311-1 (PMC8373981; doi:10.1038/s41467-021-25311-1)
Supplement: Supplementary file 1 — Supplementary Information [file 41467_2021_25311_MOESM1_ESM.pdf]

Supplementary Information

Superior Photo-carrier Diffusion Dynamics in Organic-inorganic Hybrid Perovskites Revealed by Spatiotemporal Conductivity Imaging

Xuejian Ma[†1], Fei Zhang[†2], Zhaodong Chu[1], Ji Hao[2], Xihan Chen[2], Jiamin Quan[1], Zhiyuan Huang[2], Xiaoming Wang[3], Xiaoqin Li[1], Yanfa Yan[3], Kai Zhu[2], Keji Lai[1*]

[1] Department of Physics, University of Texas at Austin, Austin, Texas 78712, USA

[2] Chemistry and Nanoscience Center, National Renewable Energy Laboratory, Golden, Colorado 80401, USA

[3] Department of Physics and Astronomy, University of Toledo, Toledo, OH 43606, USA

[†] These authors contributed equally to this work

* E-mails: kai.zhu@nrel.gov; kejilai@physics.utexas.edu

Supplementary Note 1. Solar cell device characterization

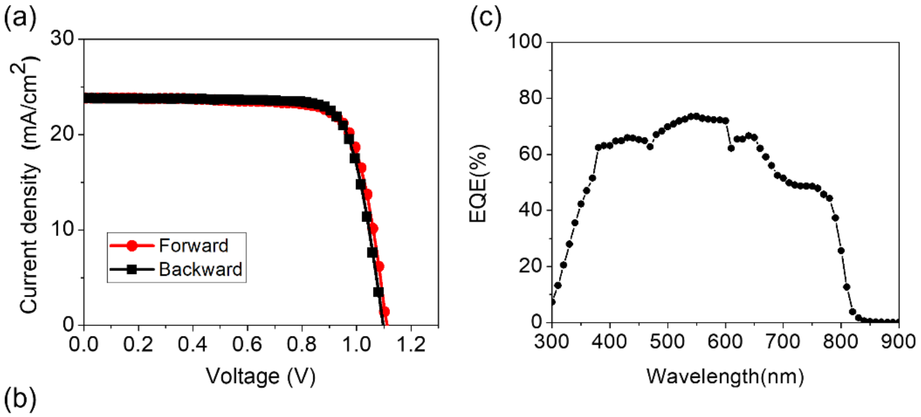

(b)

Direction	J_{SC} (mA·cm^{-2})	V_{OC}(V)	*FF*	*PCE*(%)
Forward	23.81	1.10	0.77	20.2
Backward	23.79	1.10	0.78	20.4

Supplementary Figure 1. Standard AM1.5 solar cell device performance. (**a**) I-V characteristics of the solar cell device made from the 550-nm-thick $FA_{0.95}Cs_{0.05}PbI_3$ thin films. (**b**) Table of the device parameters, listing the short-circuit current density J_{sc}, open-circuit voltage V_{oc}, fill factor (FF), and PCE of both films. (**c**) External quantum efficiency (EQE) spectra of the 250-nm-thick $FA_{0.95}Cs_{0.05}PbI_3$ thin film.

Solar cell devices using the 5% Cs-doped $FA_{0.95}Cs_{0.05}PbI_3$ thin film were prepared on conductive fluorine-doped tin oxide (FTO)-coated glass substrates. The substrates were cleaned extensively by deionized water, acetone, and isopropanol. A compact titanium dioxide (TiO_2) layer of about 40 nm was deposited by spray pyrolysis of 7-mL 2-propanol solution containing 0.6-mL titanium diisopropoxide bis(acetylacetonate) solution (75% in 2-propanol, Sigma-Aldrich) and 0.4-mL acetylacetone at 450°C in air. The $FA_{0.95}Cs_{0.05}PbI_3$ precursor solution was prepared by dissolving 1.2M Pb^{2+} in dimethyl sulfoxide (DMSO) and dimethylformamide (v/v = 3/7) mixed solvent. Perovskite films were deposited using a three-step spin-coating procedure with the first step of 100 rpm for 3 s, a second step of 3,500 rpm for 10 s, and the last step of 5,000 rpm for 30 s. Toluene (1 mL) was applied on the spinning substrates at 20 s of the third step. After spin coating, the substrates were annealed at 170°C for 27 min. The spiro-OMeTAD is prepared by the details in the HTL preparation section and deposited according to the previous report. The devices

were finalized by thermal evaporation of 100-nm gold. Solar cell performance measurements were taken under a simulated AM 1.5G illumination (100 mW/cm^2, Oriel Sol3A Class AAA Solar Simulator). The photocurrent density–voltage (J–V) characteristics were measured using a Keithley 2400 source meter. The J–V curves of all devices were measured by masking the active area with a metal mask of area 0.12 cm^2. Both backward-scan and forward-scan curves were measured with a bias step of 10 mV and delay time of 0.05 s. Typical J–V curves are shown in Supplementary Fig. 1a and the results are summarized in Supplementary Fig. 1b, for 550-nm-thick perovskite based solar cells. Supplementary Fig. 1c shows the external quantum efficiency (EQE) spectra of our 250-nm-thick $FA_{0.95}Cs_{0.05}PbI_3$ thin films measured by a solar cell quantum-efficiency measurement system (QEX10, PV Measurements).

Supplementary Note 2. Finite-element analysis of the iMIM signals

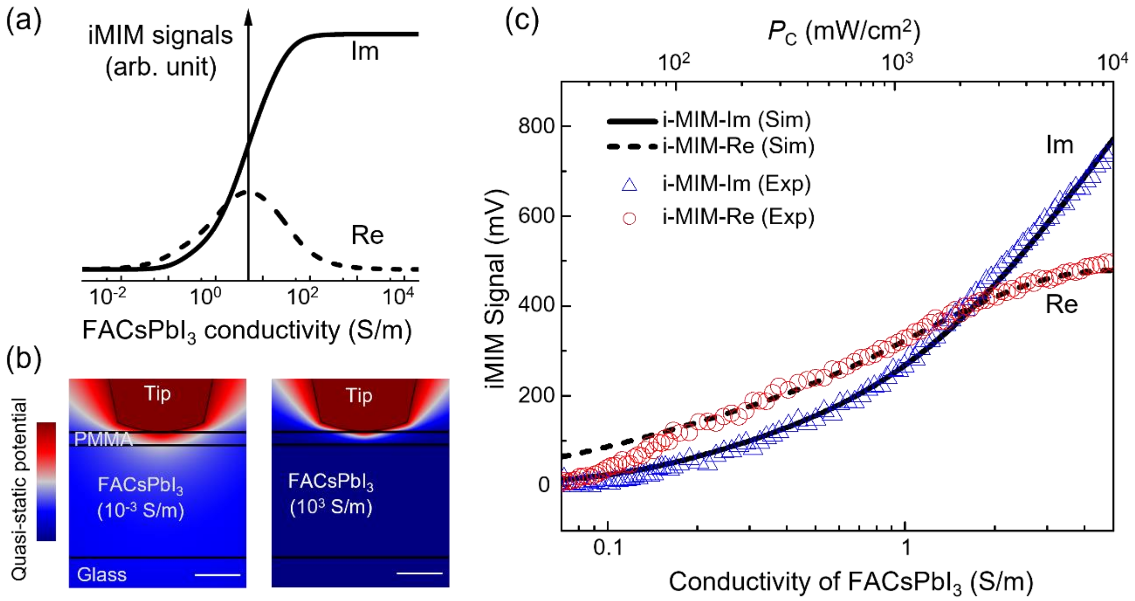

Supplementary Figure 2. Comparison between experimental data and simulation results. (**a**) Simulated iMIM response as a function of the conductivity of the perovskite layer. (**b**) Distribution of quasi-static potential near the tip apex at σ = 10^{-3} S/m (left) and 10^{3} S/m (right). (**c**) Measured (open circles and triangles) iMIM signals as a function of laser power and simulated (solid and dashed lines) results as a function of the $FACsPbI_3$ conductivity. Note that the photoconductivity does not scale with the illumination intensity.

The iMIM electronics detect the tip-sample impedance change, from which the local conductivity can be deduced. Supplementary Fig. 2a shows the finite-element analysis (FEA) results of the iMIM signals[1]. As the conductivity σ of $FACsPbI_3$ increases, the microwave electrical fields are gradually screened by the free carriers. Consequently, the imaginary part of the signal, iMIM-Im (proportional to tip-sample capacitance), increases monotonically with respect to σ, whereas the real-part signal iMIM-Re (proportional to tip-sample conductance) peaks at σ ~ 10 S/m. As depicted in the insets of Supplementary Fig. 2b, the spread of the quasistatic potential indicates that the spatial resolution of iMIM is comparable to the tip diameter. The conversion from iMIM images to conductivity images is achieved by directly comparing the

experimental data and simulated results. To this end, we performed a point measurement on Sample A and measured the iMIM signals as a function of the laser intensity. As plotted in Supplementary Fig. 2c, the response curve computed by FEA (iMIM signals versus conductivity) and the experimental data (iMIM signals versus laser power) are in excellent agreement over a wide range of parameter space[1]. We can therefore replot the raw iMIM images to photoconductivity maps with high conversion fidelity.

Supplementary Note 3. Power-dependent diffusion data for Sample A

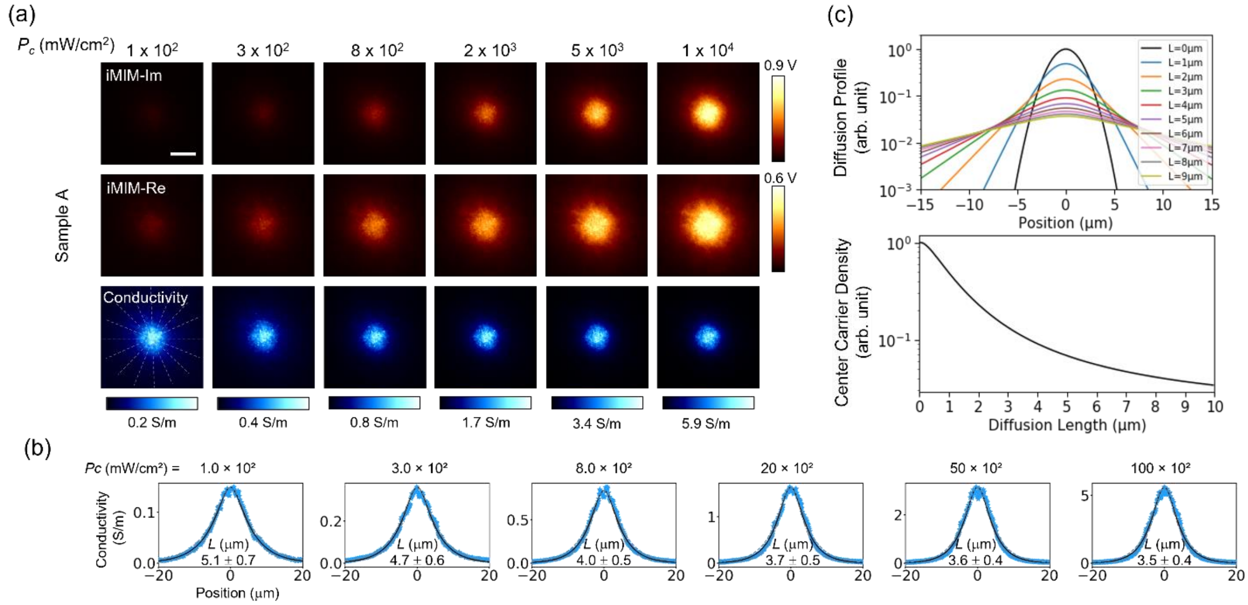

Supplementary Figure 3. Power-dependent diffusion mapping for Sample A. (**a**) Power-dependent iMIM-Im/Re and photoconductivity images of Sample A. (**b**) Line profiles of averaged photoconductivity and laser intensity and curve fittings, from which the diffusion lengths can be extracted. (**c**) (Upper panel) Spatial distribution of charge carriers with various diffusion lengths. (Lower panel) Carrier density at the center of the illumination spot as a function of diffusion length.

The power-dependent iMIM-Im/Re images of Sample A are shown in Supplementary Fig. 3a. As depicted in Supplementary Fig. 3b, the curve fitting to Eq. (3) in the main text allows us to obtain the diffusion lengths at different laser power. Using Eqs. (2) and (3) in the main text, we can quantitatively analyze the diffusion pattern. Supplementary Fig. 3c shows the spatial distribution of charge carriers with various diffusion lengths under an excitation profile e^{-r^2/w^2} with w ~ 2 μm. For instance, assuming $n_{c0} = \eta(P_c\tau/h\nu)$ is the density at the center of laser spot for L = 0 μm, the numerical solution indicates that n_c ~ 0.07 n_{c0} for L = 5 μm [2]. The analysis is important for the calculation of carrier density and mobility, which are tabulated in Fig. 3d.

Supplementary Note 4. Finite element analysis for the ultra-sharp tip.

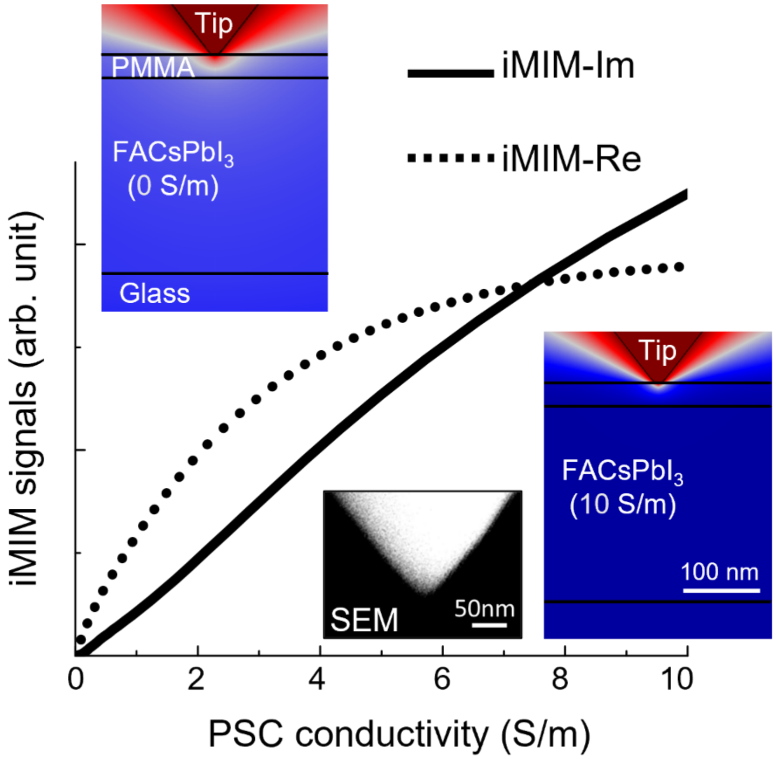

Supplementary Figure 4. Simulated iMIM response for the ultra-sharp tip as a function of the conductivity. Distributions of quasi-static potential are shown at σ = 0 S/m (left) and 10 S/m (right).

For the point experiment shown in Fig. 2 of the main text, we used an ultra-sharp tip from Rocky Mountain Nanotechnology LLC, model 12PtIr400A-10 with a nominal radius of 10 nm at the apex, for accurate positioning on grains and grain boundaries. For the FEA modeling, we have used the half-angle of 38° and a diameter of 20 nm at the apex that are consistent with the SEM image. As shown in Supplementary Fig. 4, the simulated iMIM-Im signals roughly scales with the conductivity of $FACsPbI_3$ up to σ ~ 10 S/m, which is within the photoconductivity range in our experiment. As a result, we directly analyze the tr-iMIM-Im signals rather than converting them to photoconductivity in the time-resolved response.

Supplementary Note 5. Wavelength-dependence of the tr-iMIM data

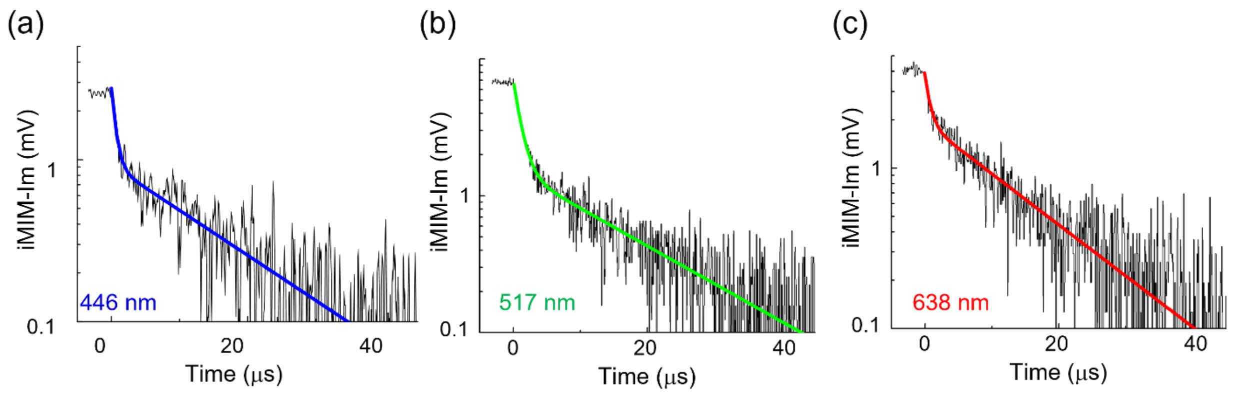

Supplementary Figure 5. Wavelength-dependent tr-iMIM-Im signals with laser intensity ~ 100 mW/cm^2.

The majority of data presented in the main text were acquired under the illumination of a 446-nm diode laser. We also performed tr-iMIM experiments with two additional diode lasers with wavelengths of 517 nm (green) and 638 nm (red). As shown in Supplementary Fig. 5, the two relaxation time constants in Sample A were observed in all three measurements. It is possible that the thermalization to the band edge through phonon emitting is very effective at low laser intensities (~ 1 Sun), after which the trapping/detrapping processes take place. As a result, the carrier temperature is essentially independent of the excitation wavelength. We therefore conclude that the two lifetimes are intrinsic for the $FACsPbI_3$ thin films.

Supplementary Note 6. Complete AFM/iMIM data in the sample-scan mode

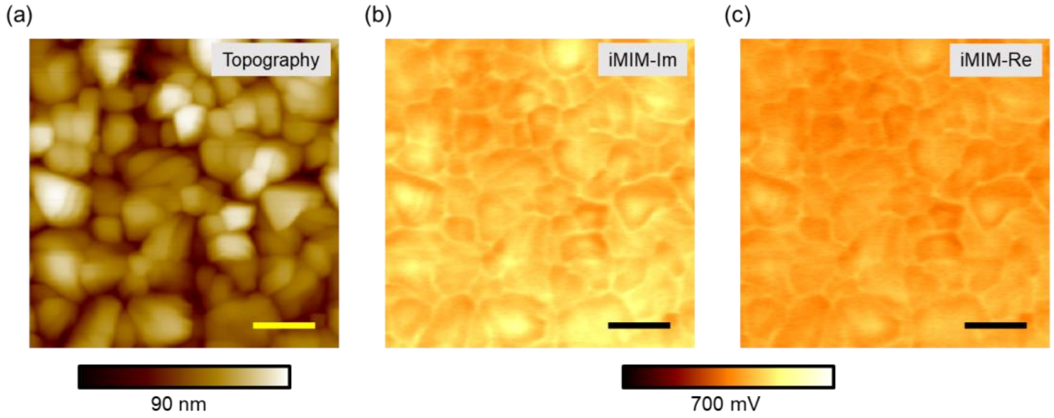

Supplementary Figure 6. Raw AFM and iMIM-Im/Re images of sample A. (**a**) AFM, (**b**) iMIM-Im, (**c**) iMIM-Re images. All scale bars are 4 μm.

The raw AFM and iMIM-Im/Re images are shown in Supplementary Fig. 6. Note that the contrast in the iMIM images is mostly due to topographic crosstalk[3].

Supplementary Note 7. Complete tr-iMIM data for grains and grain boundaries

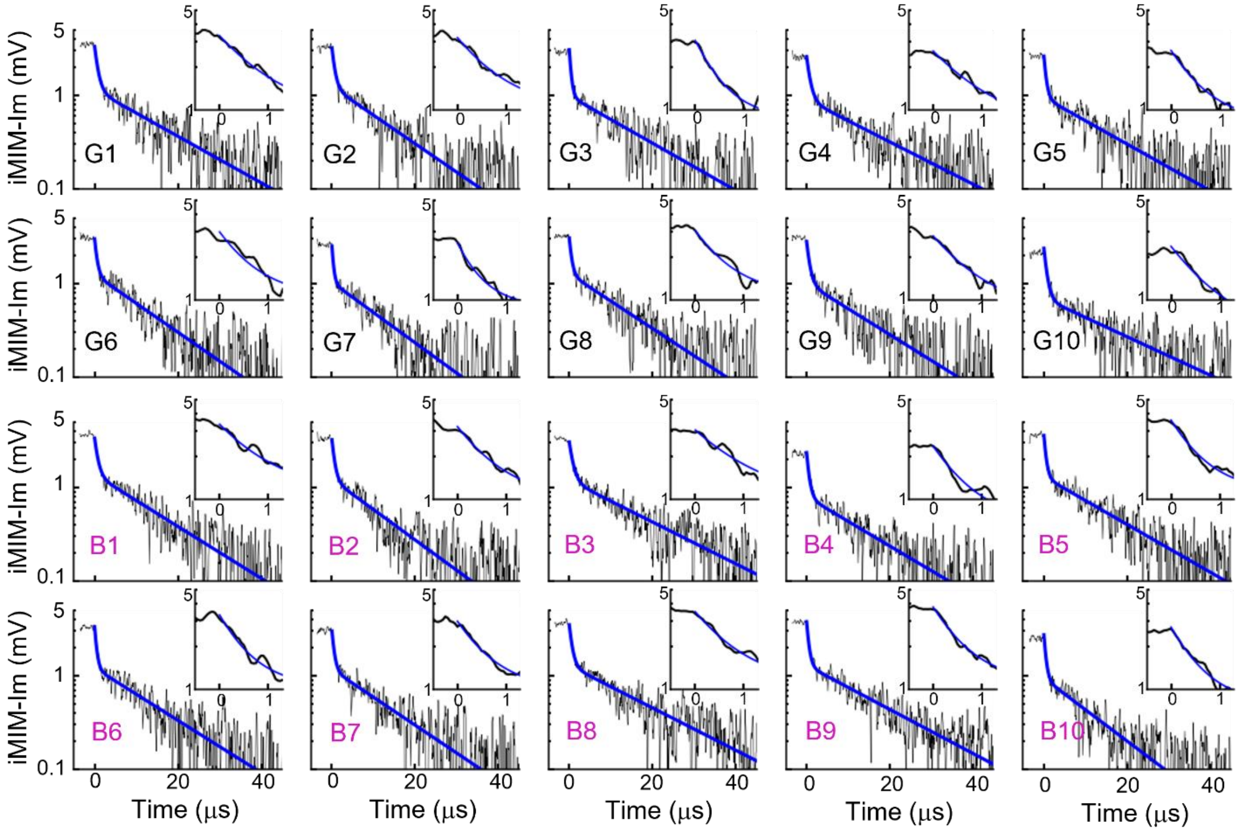

Supplementary Figure 7. Complete set of tr-iMIM-Im data for 20 points. G1-G10 are points on grains, while B1-B10 are points on grain boundaries.

The complete set of tr-iMIM data and biexponential fitting are included in Supplementary Fig. 7. It is evident from the raw data that the temporal dynamics of photo-carriers are spatially uniform across microstructures on the $FACsPbI_3$ sample surface.

Supplementary Note 8. PL measurements on Samples B and C

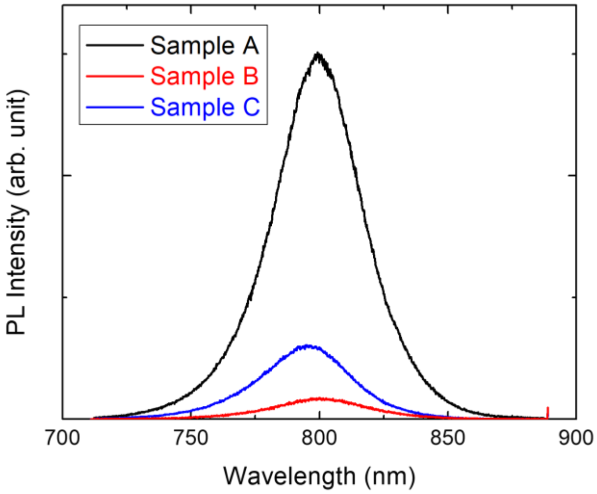

Supplementary Figure 8. PL data on Samples A, B, and C at an excitation laser power of 9.6×10^3 mW/cm^2

We have conducted photoluminescence (PL) measurements on Samples A, B, and C to confirm the charge transfer in HTL/ETL samples. As shown in Supplementary Fig. 8, the PL signal is quenched by 94% in the PTAA/$FACsPbI_3$ Sample B and 80% in the TiO_2/$FACsPbI_3$ Sample C. It should be noted that, for good signal-to-noise ratio, the excitation power (9.6×10^3 mW/cm^2, on the order of 100 Sun) is kept high in the PL experiments. The incomplete PL quenching in Samples B and C is consistent with the high-power tr-iMIM data in Fig. 4.

Supplementary Note 9. Tabulated mobility values for MAPI/FAPI thin films

Composition	Measurements	μ_e (cm^2/V·s)	μ_h (cm^2/V·s)	Suppl. Ref.
$MAPbI_3$	TRMC	12.5	7.5	5
$MAPbI_3$	TRMC	3	17	6
$MAPbI_3$	TRPL	1.4	0.9	7
$MAPbI_3$	TRPL	0.7	0.4	8
$MAPbI_3$	TRTS	2.32	26	9
$(FA,MA)Pb(I,Br)_3$	TRTS	0.234	17.5	9
$(FA,MA,Cs)Pb(I,Br)_3$	TRTS	1.12	29.1	9
$MAPbI_3$	TRMC	30.5	6.5	10
$MAPbI_3$	TRMC	18	2.5	11
$FAPbI_3$	TRPL	0.2	3.5	12
$MAPbI_3$	Transport	~1	~0.2	13

Supplementary Figure 9. Tabulated mobility values for MAPI/FAPI thin films. Reference numbers are taken from the list in the main text. Abbreviations: TMRC – Time-resolved microwave conductivity, TRPL – time-resolved photoluminescence, TRTS – time-resolved THz spectroscopy.

Reported mobility values for MAPI/FAPI thin films are shown in Supplementary Fig. 9. There is no consensus on whether electrons or holes have the higher mobility in the organometal trihalide family. Mobility values measured by the same methods from different groups may also show opposite results, suggesting a strong sample dependence of scattering mechanisms. It is worth noting that, as acknowledged by the authors, the two measured mobilities from Ref. 17 (highlighted in blue) are not unambiguously associated with electrons or holes based on the TRTS method. Instead, the higher one was assigned to holes and lower one to electrons purely based on assumptions from previous reports[4].

Supplementary Note 10. Complete tr-iMIM data of Sample B and Sample C

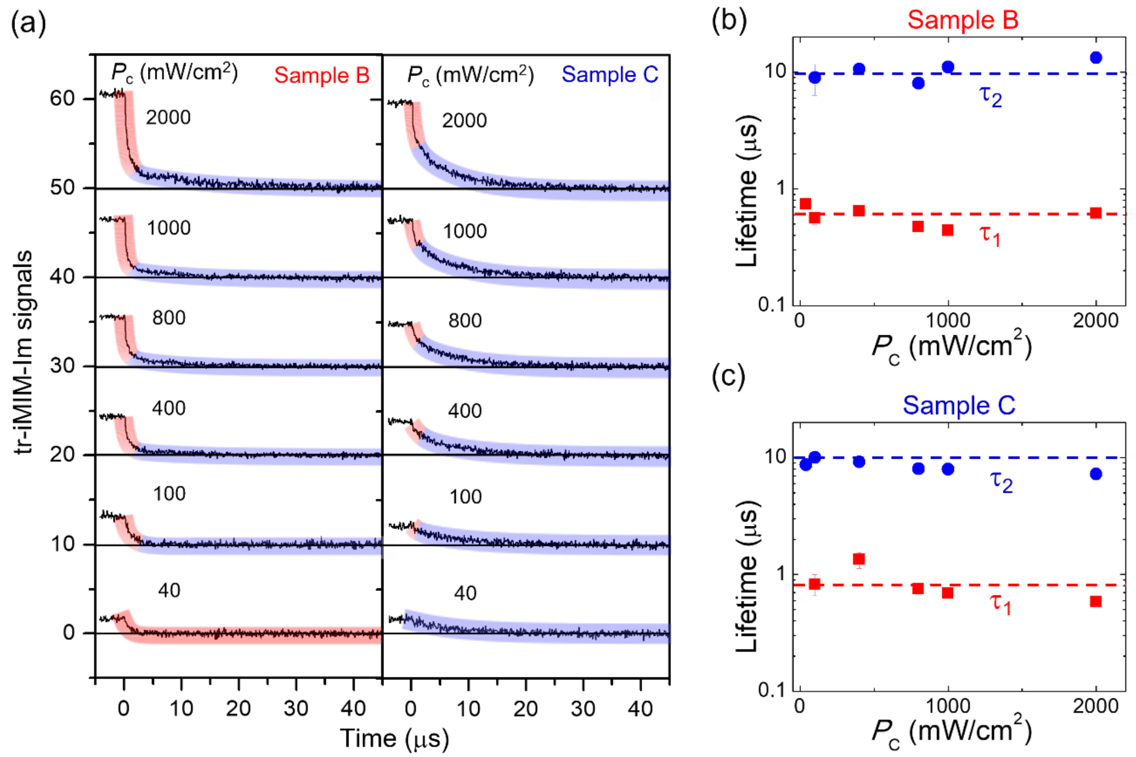

Supplementary Figure 10. Complete tr-iMIM data of sample B and C. (**a**) Complete tr-iMIM data in linear scale without truncation. The thick lines shaded in red and blue cover the segments of data with fast and slow decays, respectively. (**b**) Power-dependent lifetimes for Sample B. (**c**) Power-dependent lifetimes for Sample C.

The complete tr-iMIM data (without truncating data below the noise floor in Figs. 4a and 4b of the main text) of Sample B and Sample C are shown in Supplementary Fig. 10a. For the two samples, the power-dependent time constants extracted from biexponential fitting are plotted in Figs. S10b and S10c, respectively. The two lifetimes τ_1 ~ 0.7 μs and τ_2 ~ 10 μs are consistently observed in both samples.

Supplementary Note 11. Complete diffusion mapping data for Sample B

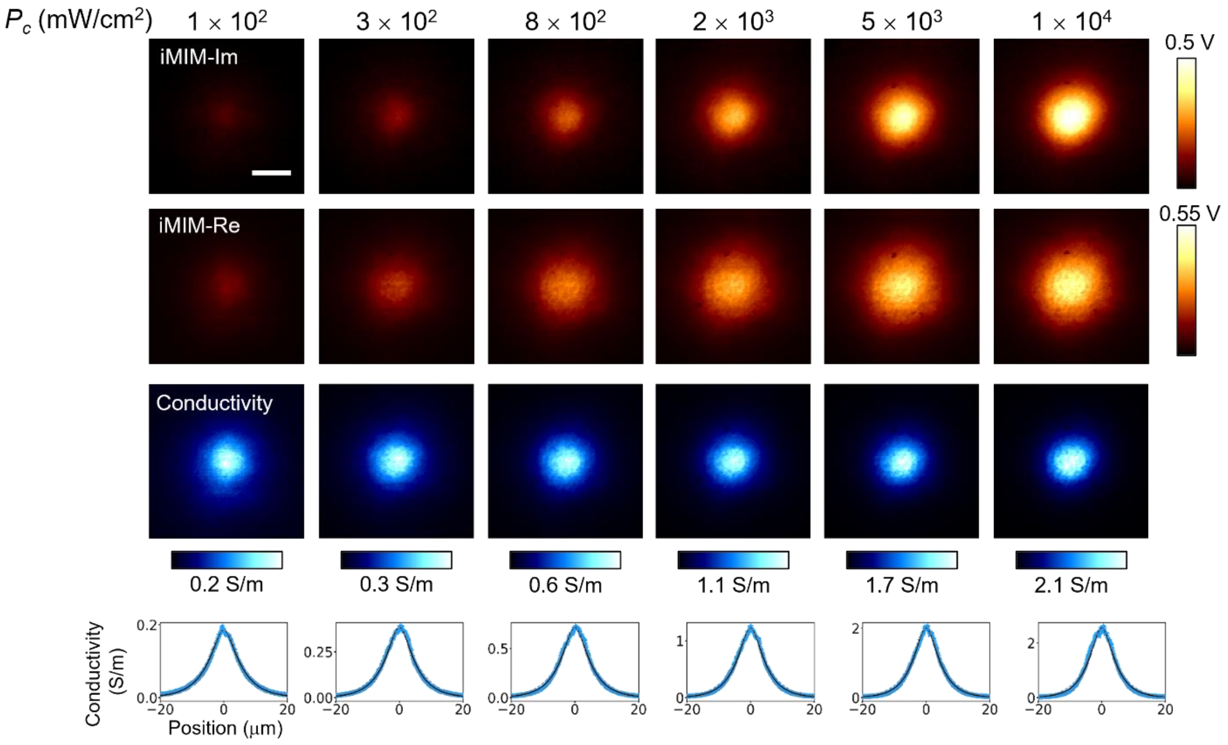

Supplementary Figure 11. Analysis of diffusion images for Sample B. (Top) The raw iMIM-Im, iMIM-Re, and conductivity images. (Bottom) Averaged converted conductivity linecuts for Sample B.

Supplementary Fig. 11 shows the raw iMIM-Im/Re and conductivity images for diffusion mapping in Samples B. Curve fittings to Eq. (3) in the main text are also plotted in the figure.

Supplementary Note 12. Complete diffusion mapping data for Sample C

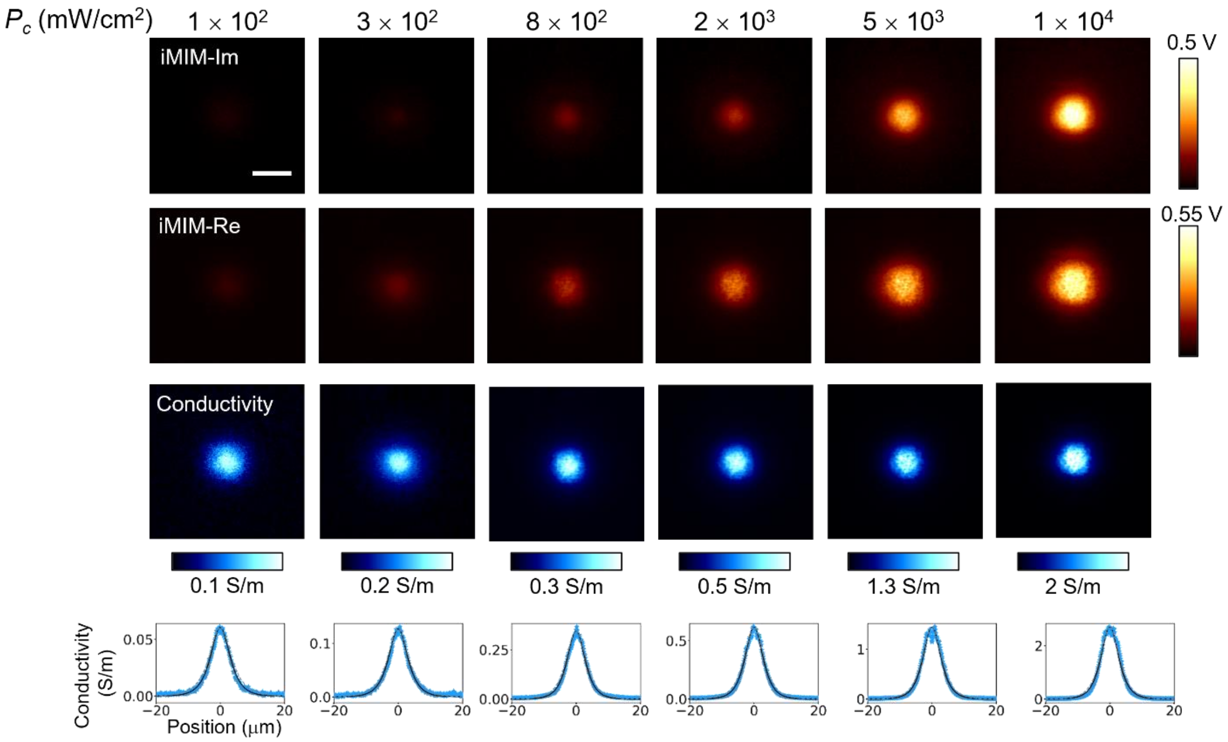

Supplementary Figure 12. Analysis of diffusion images for Sample C. (Top) The raw iMIM-Im, iMIM-Re, and conductivity images. (Bottom) Averaged converted conductivity linecuts for Sample C.

Supplementary Fig. 12 shows the raw iMIM-Im/Re and conductivity images for diffusion mapping in Samples C. Curve fittings to Eq. (3) in the main text are also plotted in the figure.

Supplementary References:

1. Lai, K., Kundhikanjana, W., Kelly, M., and Shen, Z. X. "Modeling and characterization of a cantilever-based near-field scanning microwave impedance microscope", *Rev. Sci. Instrum*. **79**, 063703 (2008).
2. Chu, Z. et al. "Unveiling defect-mediated carrier dynamics in monolayer semiconductors by spatiotemporal microwave imaging", *Proc. Natl. Acad. Sci.* **117**, 13908–13913 (2020).
3. Chu, Z. et al. "Impact of grain boundaries on efficiency and stability of organic-inorganic trihalide perovskites", *Nat. Commun.* **8**, 2230 (2017).
4. Zhai, Y. et al. "Individual Electron and Hole Mobilities in Lead-Halide Perovskites Revealed by Noncontact Methods", *ACS Energy Lett.* **5**, 47−55 (2020).
5. Ponseca, C. S. et al. "Organometal halide perovskite solar cell materials rationalized: ultrafast charge generation, high and microsecond-long balanced mobilities, and slow recombination", *J. Am. Chem. Soc*. **136**, 5189–5190 (2014).
6. Oga, H., Saeki, A., Ogomi, Y., Hayase, S., and Seki, S. "Improved understanding of the electronic and energetic landscapes of perovskite solar cells: high local charge carrier mobility, reduced recombination, and extremely shallow traps", *J. Am. Chem. Soc*. **136**, 13818–13825 (2014).
7. Stranks, S. D. et al. "Electron-hole diffusion lengths exceeding 1 micrometer in an organometal trihalide perovskite absorber", *Science* **342**, 341–344 (2013).
8. Xing, G. et al. "Long-Range Balanced Electron-and Hole-Transport Lengths in Organic-Inorganic $CH_3NH_3PbI_3$", *Science* **342**, 341–347 (2013).
9. Zhai, Y. et al. "Individual Electron and Hole Mobilities in Lead-Halide Perovskites Revealed by Noncontact Methods", *ACS Energy Lett*. **5**, 47−55 (2020).
10. Hutter, E.M. et al. "Charge Transfer from Methylammonium Lead Iodide Perovskite to Organic Transport Materials: Efficiencies, Transfer Rates, and Interfacial Recombination", *Adv. Energy Mater*. **7**, 1602349 (2017).
11. Levine, I. et al. "Can we use time-resolved measurements to get steady-state transport data for halide perovskites?", *J. Appl. Phys*. **124**, 103103 (2018).
12. Eperon, G.E. et al. "Formamidinium lead trihalide: a broadly tunable perovskite for efficient planar heterojunction solar cells", *Energy Environ. Sci*. **7**, 982 (2014).

13. Castro-Méndez, A.F., Hidalgo, J., and Correa-Baena, J.P. "The Role of Grain Boundaries in Perovskite Solar Cells", *Adv. Energy Mater*. **9**, 1901489 (2019).
